# “It feels like I’m coming to a friend’s house”: an interpretive descriptive study of an integrated care site offering iOAT (Dr. Peter Centre)

**DOI:** 10.1186/s13722-023-00428-4

**Published:** 2023-12-02

**Authors:** Sophia Dobischok, José R. Carvajal, Kyle Turner, Kaitlyn Jaffe, Eisha Lehal, Sarinn Blawatt, Casey Redquest, Rosalind Baltzer Turje, Patrick McDougall, Bryce Koch, Cheryl McDermid, Damon Hassan, Scott Harrison, Eugenia Oviedo-Joekes

**Affiliations:** 1 Centre for Advancing Health Outcomes, Providence Health Care, St. Paul’s Hospital, 575- 1081 Burrard St., Vancouver, BC V6Z 1Y6 Canada; 2 Department of Education and Counselling Psychology, McGill University, 3700 McTavish St., Montreal, QC H3A 1Y2 Canada; 3 Department of Health Promotion and Policy, University of Massachusetts Amherst, 715 North Pleasant Street, Amherst, MA 01003 USA; 4 Dr. Peter Centre, 1110 Comox Street, Vancouver, BC V6E 1K5 Canada; 5 Providence Health Care, Providence Crosstown Clinic, 77 East Hastings Street, Vancouver, BC V6B 1G6 Canada; 6 School of Population and Public Health, University of British Columbia, 2206 East Mall, Vancouver, BC V6T 1Z3 Canada

## Abstract

**Background:**

Injectable opioid agonist treatment (iOAT) has proven to be a safe and effective treatment option for severe opioid use disorder (OUD). Yet, iOAT is often isolated from other health and social services. To align with a person-centered care approach, iOAT can be embedded in sites that combine systems and services that have been historically fragmented and that address multiple comorbidities (integrated care sites). The present study investigates the addition of iOAT at an integrated care in Vancouver, British Columbia. We aimed to capture what it means for service users and service providers to incorporate iOAT in an integrated care site and describe the processes by which the site keeps people engaged.

**Methods:**

We conducted 22 interviews with 15 service users and 14 interviews with 13 service providers across two rounds of individual semi-structured interviews (Fall 2021, Summer 2022). The second interview round was precipitated by a service interruption in medication dispensation. Interview audio was recorded, transcribed, and then analysed in NVivo 1.6 following an interpretive description approach.

**Results:**

The emergent themes from the analysis are represented in two categories: (1) a holistic approach (client autonomy, de-medicalized care, supportive staff relationships, multiple opportunities for engagement, barriers to iOAT integration) and (2) a sense of place (physical location, social connection and community belonging, food).

**Conclusion:**

Incorporating iOAT at an integrated care site revealed how iOAT delivery can be strengthened through its direct connection to a diverse, comprehensive network of health and social services that are provided in a community atmosphere with high quality therapeutic relationships.

**Supplementary Information:**

The online version contains supplementary material available at 10.1186/s13722-023-00428-4.

## Introduction

An estimated 16 million people globally live with the severe effects of Opioid Use Disorder (OUD) [15]. In British Columbia, the Canadian region hardest hit by the overdose crisis, over 10,000 people have died since the crisis was declared in 2016 [26]. Injectable opioid agonist treatment (iOAT) is one possible OUD treatment option that has been shown in multiple clinical trials to be safe, effective [16, 24, 32, 40, 48], and economically feasible [3, 37]. IOAT (e.g., with diacetylmorphine, hydromorphone) is typically dispensed to clients under direct clinical observation for intravenous or intramuscular self-administration. Most clients must attend the treatment site in-person up to three times a day for their injections, while a small percentage of clients have prescriptions for take-home doses [38, 39]. The daily clinic visits provide opportunities for therapeutic relationship building between iOAT clients and service providers, and engagement in other addiction care services [4, 41]. At the same time, the rigid protocols and supervision render iOAT a demanding and highly medicalized option that does not align with core principles in person-centered addiction care such as autonomy, individualized treatment, and holistic care [33]. To attract clients with diverse needs who could benefit from this treatment, iOAT can be expanded beyond isolated clinical sites and situated within the broader scope of person-centered addiction care.

One way to provide iOAT within a person-centered care approach is to embed the service within an integrated care site. Integrated care means bringing together systems and services that have been historically fragmented and that address multiple comorbidities, such as by amalgamating clients’ physical and psychosocial care at one treatment site [11, 22, 27]. Prior research has identified pharmacological therapy, psychosocial services, and education/outreach as the most needed integrated services for OUD treatment [29], and many OUD clients hold positive perceptions of integrated care [45]. While integrated care sites have similar client retention rates to other care approaches, they can capture clients whom the other programs may not be able to retain [29]. Most Canadian iOAT sites do not classify as full integrated care, as their resources are only equipped to accommodate a portion of the ancillary services OUD clients may require (e.g. medical care, outreach workers, nutrition) and must otherwise refer clients out to the community for their other needs [18]. Having iOAT splintered from other needed services increases the barrier for clients to access a full breadth of care.

The Doctor Peter Centre (DPC) is an integrated care site in Vancouver, BC that offers comprehensive wrap-around services to support the known structural vulnerabilities present in the population they serve [11]. The DPC primarily serves people living with HIV, a group that frequently experiences comorbid intersecting health and social issues including Hepatitis C, substance use disorders, illicit drug use, trauma, unstable housing, and/or poverty [2]. The DPC practices a holistic approach to healthcare: beyond medical services (e.g., primary care, wound care, medication provision), they provide two nutrient dense meals a day, individual and group counselling, housing assistance, access to showers and personal hygiene products, recreation therapy, and supervised injection services (among other services) [21]. The DPC’s integrated care approach has previously been associated with improved health outcomes, such as increased likelihood of viral load suppression in HIV treatment [52].

To engage their members who live with OUD in the continuum of care, the DPC provides opioid agonist treatment (OAT) medications such as methadone and buprenorphine. In 2019, the DPC began offering iOAT with diacetylmorphine (DAM) or hydromorphone (HDM) to better serve their members who were not engaged or retained by traditional OAT [25]. The implementation of iOAT at the DPC presents a unique opportunity to investigate the provision of iOAT within an integrated, person-centered care model. As part of the Program of Outcomes Research on Treatment with Injectables for Addiction (PORTIA) study, we conducted an exploratory qualitative study that aimed to (1) investigate what role the integration of iOAT plays on service users’ wellness trajectory within an integrated care site and the broader context of OUD treatment and harm reduction; (2) investigate what dynamics emerge between service users, the treatment, the site, and the broader context that may impact service delivery and service users’ continuation of care. These findings can aid care providers, policy makers, and community champions across Canada who aim to implement iOAT in new ways and advance evidence-based policies for OUD.

## Methods

### Study setting

The DPC opened in 1997 in Vancouver’s West End neighbourhood as an integrated care site and social space for people living with HIV to receive clinical and social services, access recreation activities, and interact with peers without stigma. Services offered at the site include the Day Health Program (e.g., daily medication dispensation, opioid maintenance treatment, nutrient dense meals, supervised injection rooms, showers, sleeping rooms, counselling, art, music, and recreation therapies), 24-hour specialized nursing care residence, and enhanced supportive housing for residents well enough to transition out of specialized nursing care residence. Most individuals who arrive for stabilization services are referred from the community, although many are transferred directly from hospital [2]. Members at this site live with a constellation of HIV, Hepatitis C, trauma, histories of unstable housing, poverty, and/or substance use disorders 2]. The DPC is predominantly for individuals living with HIV, and many members have histories of injection drug use, including with opioids [11, 23]. The DPC has offered iOAT with hydromorphone (HDM) and later with diacetylmorphine (DAM) since 2019, supported by Health Canada’s Substance Use and Addiction Program. Prior to this integration, clients needed an HIV diagnosis to be a DPC service user. However, admittance criteria were expanded to clients without HIV for iOAT services, drawing new individuals to the DPC. As of 2022, the DPC had 20 active iOAT service users. The DPC is one of the first Canadian organizations to incorporate iOAT within a fully integrated care site.

### Design and participants

The present exploratory qualitative study used an interpretive description approach, a qualitative methodology informed by clinical expertise and grounded in the lived experienced of people accessing clinical services, that aims to meaningfully inform clinical practice in real-time [50]. The research team collaborated with the service providers at the site to identify important aspects of their iOAT integration and consolidated these lines of inquiry into research questions and an interview guide that evolved iteratively throughout the study (see Additional file 1: Appendix S1). For example, the second interview round investigated a temporary iOAT medication transition that occurred after the initial interviews. The flexibility of interpretive description facilitates the inclusion of varied techniques carried over from complementary methodologies, such as theoretical and purposive sampling, which were used in the present study [50].

To recruit participants, on-site staff distributed our study information via word of mouth, posters, and recruitment cards. Recruitment initially focused on service users who were actively receiving iOAT at the DPC and service providers with a direct role in iOAT. In the second interview round, eligibility criteria broadened to encompass service users who previously accessed iOAT at the DPC, and service providers who did not have a direct role in iOAT service delivery, but who interacted with iOAT service users. Service providers interviewed served a diverse set of roles, from front-line health and recreation therapy staff to leaders and directors. Service users interviewed were largely older white males (two participants identified as both white and Indigenous), born in Canada with Grade 11 high school education or above. Despite our recruitment efforts, no former clients or female clients participated. Former clients are historically challenging to reach in the community as they no longer access the sites where the research team could engage them during routine access to care. At the time of interviewing, there were no current female iOAT clients at the site.

While DPC staff assisted with recruitment, data collection, and interpreting how results inform future research, they had no access to study data and were not involved in any form of data analysis or generation of results. The research team operated independently to maintain academic integrity.

### Data collection

We conducted 22 interviews with 15 service users and 14 interviews with 13 service providers (see Table 1) across two rounds of individual semi-structured interviews (Fall 2021, Summer 2022). In each round, recruitment and interviewing occurred until data reached saturation. All interviews were conducted virtually via Zoom or at the integrated care site (DPC). The virtual interviews were done to accommodate ongoing COVID-19 pandemic restrictions and service providers’ work schedules. Interview audio was recorded on two devices, stored in a secure server, de-identified, and transcribed verbatim by a third-party Canadian transcription service. Service user interviews lasted 20 to 60 min, and service provider interviews lasted over 60 min. Service users were compensated $30 per hour or fraction. Service providers were offered $60 per hour or fraction; most declined to be compensated or stated that they would donate the compensation to the center.

From September 2021 to December 2021 (round one), we interviewed 11 of the approximately 20 active iOAT service users and 6 service providers (Table 1). From May to August 2022 (round two), we interviewed 11 out of the approximately 14 active iOAT service users and eight service providers. Seven service users and one service provider participated in both interview rounds. The second round was conducted due to a service interruption in May 2022 wherein service users receiving DAM were faced with a forced and temporary two-week transition to HDM due to a change in the pharmacy providing medication to the site. Service users were given the choice to switch to injectable HDM at the DPC, or travel to another community clinic to receive injectable DAM. The medication transition was a significant and unanticipated event, as iOAT clients often have a strong preference for their medication of choice and might prioritize medication type over other features of a site. The disruption thus presented a unique opportunity to investigate service users’ unique tie to iOAT within an integrated care setting, as they might be willing to accept a non-preferred medication to remain at the site for its other features (i.e., comprehensive care, relationships with staff). Of the 11 service users interviewed in the second round, seven rotated to HDM, three were already in HDM, and one service user continued receiving DAM at a different community iOAT clinic.

**Table 1:** Rounds of interviews and number of participants

Interviews rounds	Service users ^a^	Service providers ^b^
Round 1 Only^c^	4	5
Round 2 Only^d^	4	7
Round 1 and 2	7	1
Total of participants	15	13
Total of interviews	22	14

^a^Service Users are defined as people who access iOAT at the DPC

^b^ Service Providers are defined as DPC staff, which includes individuals who or may not be directly involved in the provision of iOAT

^c^ Round 1 occurred between September 2021 to December 2021

^d^ Round 2 occurred between May 2022 to August 2022

After the interview, participants were invited to join the larger Program of Outcomes Research of Treatment with Injectables for Addiction (PORTIA) study, within which the current study is embedded. Service users who accepted the invitation completed the PORTIA questionnaire package, which includes a socio-demographic questionnaire (Table 2) and other measures of substance use, health, treatment, and psychosocial domains. Nine service providers also agreed to be interviewed and completed a separate sociodemographic form (Table 2).

**Table 2:** Self-reported service user (N = 11) and Provider (N = 9) socio-demographics

Participants	Service users N (%)	Providers N (%)
Age (M ± SD)	57.36 (± 10.16)	52.44 (± 12.84)
Race		
White	11 (100)	8 (89)
Indigenous (First Nations, Métis, or Inuk (Inuit))	2 (18)	
Other^a^		1 (11)
Born in Canada	11 (100)	8 (89)
Gender		
Male	11 (100)^b^	5 (56)
Female		4 (44)
Education		
Up to Grade 11	2 (18)	
Grade 12	6 (55)	
Some College/University	3 (27)	
Disabled	5 (45)	
Housed ^c^	11 (100)	

^a^ E.g., Black, South Asian, etc

^b^ The researchers were only able to engage with men who were accessing iOAT. Attempts that were made to engage with women who were accessing iOAT were unsuccessful

^c^ Not all participants were housed at the DPC

The study holds behavioural ethics approval from the Providence Health Care Research Ethics Board in partnership with Fraser Health Authority [H19-00217] and all participants provided voluntary written informed consent.

### Data analysis

The qualitative data were coded and analyzed following Thorne’s interpretive description approach, a method of qualitative analysis conceptualized in nursing research [49, 51]. Interpretive description allows for the incorporation of existing clinical and experiential knowledge to inform analysis and interpretation. Thus, in the conduct of thematic analysis [6], [7], analytic themes emerged from the data guided by participants’ responses and insight and informed by researcher and clinician expertise.

NVivo 1.6 was used to manage, organize, and analyze the data. While interviews were conducted in two rounds, coding and analysis were conducted cross-sectionally, given the specific emphasis on understanding the impacts of service interruption in Round 2. Service user and provider interviews were assessed for initial themes independently of one another. Initial data coding was completed independently by two research assistants. The research team then agreed upon an initial set of broad codes. The codes were used to identify patterns across the dataset. The research team met on a regular basis to discuss the evolving codebook. Codes indicating thematic similarities between several interviews were grouped and summarized into preliminary themes which were discussed and analyzed relationally within the research. Constructing themes took several drafts, and was informed by the source data, the codebook, and contextual data (e.g., memos and observational descriptions the team collected during data collection). Code names and summaries were labelled according to recurring words/phrases from interviews. A final report based on these descriptive summaries was framed, and illustrative quotations from the transcripts were chosen to underpin selected themes [49]. Findings from this report were shared with stakeholders.

## Results

**Fig. 1:**
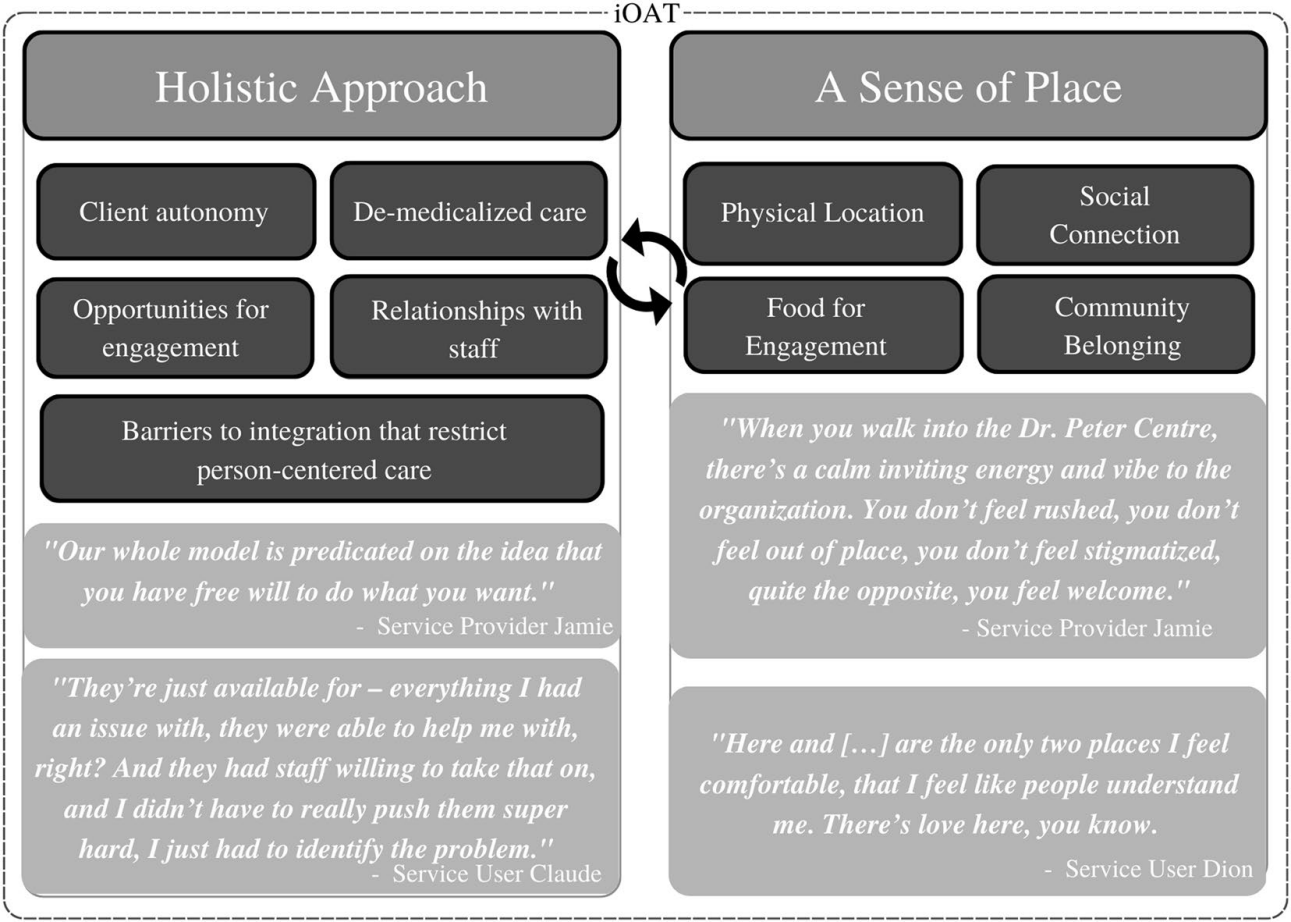
Emerging themes on incorporating iOAT into an integrated care site

Our study aimed to capture what it means for service users and service providers to incorporate iOAT in an integrated care/services site and explore the processes by which the site facilitates engagement. The main emerging themes are represented in two categories: (1) a holistic approach (client autonomy, de-medicalized care, supportive staff relationships, multiple opportunities for engagement, barriers to iOAT integration) and (2) a sense of place (physical location, social connection and community belonging, food) [19, 34]) (Fig. 1). All service user and provider names in the results section are aliases to protect anonymity.

### A holistic approach

The following themes are elements of the DPC’s holistic, person-centered care (PCC) approach that both service users and service providers cited as processes which contributed strongly to the integration of iOAT at the site and in the lives of the participants. We thus frame the following factors as holistic in that they relate to how the incorporation of iOAT aligns with the DPC’s overarching philosophy of attending to the diverse, individualized needs of the whole person.

#### Multiple opportunities for engagement

Beyond iOAT, this site offers a diverse range of services that service users and providers described as meaningful engagement opportunities. Service users thus situated their enrolment in iOAT not just as an isolated medical treatment, but as being embedded in a wide network of support systems to their overall well-being. For instance, William, who accesses iOAT, describes the comprehensive services offered at DPC that improve his wellbeing:

*And, you know, there’s counselling if you need help with whatever emotional issues you have and whatever. There’s a music therapy program; there’s instruments galore. I was just playing the piano here before I came. Computers. You know? Access to the internet. You have a weight room. You know? Like, it’s a mini gym there. You know? The list goes on and on.*

For service providers, offering multiple services at one site lessens the burden on clients to move between numerous sites to access the necessary services for their medical care and personal wellbeing.

*…you have to be [at iOAT] two or three times a day. And it’s just so much more beneficial in my humble estimation to be able to go to a place where you’ve got your breakfast, then you take your morning meds, you take your iOAT, and then even if you go off you sort of included other activities of daily living in that rather than having to go somewhere and pick up your breakfast then you have to go somewhere else and pick up your meds from the pharmacy. Then you’ve got to and get your iOAT and then by the time you’ve done that you’re thinking of lunch and then you have to go somewhere to get your meal and then you have to then go back to iOAT.* (Service Provider Riley)

By having multiple services in one location, the DPC thus has multiple points of entry into care and streamlines service users’ ability to access more than one necessary service.

#### Client autonomy in treatment engagement

A core element of PCC in addiction care is autonomy [33]. Participants (both service users and providers) described how the DPC offers service users autonomy in how much or little they engage with the site, and what types of services they engage with. Specifically, clients have numerous opportunities to engage at the integrated care facility in addition to their iOAT care, as Riley describes above, yet they are under no pressure or obligation to engage. This means that each service user accessing iOAT can have a unique, self-determined level of involvement that aligns with their individual needs and preferences:

*If you’re a client at the Dr. Peter Centre, you can come in and you can just come in, have food and leave. You can come in, have food, get your medication, you can come in and stay all day and leave, you can do whatever you want. Our whole model is predicated on the idea that you have free will to do what you want.* (Service Provider Jamie)

*I really kicked myself over the years, because – the services - I haven’t availed myself enough. The music programme, art programme. And, you know, lots of social stuff too, like karaoke and music sessions. They do a lot, but you have to want to access it. I don’t access much of it I just keep to myself. So it’s a great place, and when I have the time I’d want to get more involved in what they have.* (Service User Darryl).

Ultimately, the power to choose their level of involvement within the range of service options at this integrated care site supports clients to determine their own individualized trajectory of care without system-level pressures on what care should look like.

#### De-medicalized care

IOAT can be a highly medicalized treatment characterized by regulations, protocols, and monitoring. The medicalization may act as a barrier to service users who have experienced trauma in clinical/medical settings, or who feel reluctant to engage with a medical setting multiple times a day, every day. Despite the potential for iOAT to shift the integrated care site to a more clinical atmosphere, service users continued to describe the site as a comfortable, welcoming, non-medical space. For example, the injection room was decorated based on service users’ input, including requested art and posters. Service providers affirmed that the site intentionally cultivates a community atmosphere that is distinct from other healthcare settings where the service users may have experienced trauma. A service provider, Drew, explains the rationale behind this design:

*They are not really familiar with as well as they are somewhat afraid of going* [into healthcare settings] *because of their trauma from the system. We try to break down all of those things that – as you can see, I don’t really wear any kind of uniform or scrubs that they can be a little bit afraid of. So, we kind of try to make it here, at the Dr. Peter Centre, their secondary community, so that they can be safe and they can feel safe enough to socialize themselves and make a little society on their own. So, I think that’s the – I think the biggest differences between the Dr. Peter Centre*.

Leo, a service user, similarly described the community-like feeling of the DPC:

*People here have been through the same journeys. Like I – never once – when I come to the iOAT place, to get my medication, I don’t feel like I’m coming to a doctor’s office. It doesn’t feel that way. It feels like I’m coming to a friend’s house. It does. It feels like that to me that they’ve never been judgemental towards anything that I’ve done and that’s rare.*

The de-medicalized approach to care allows service users to experience the integrated care site as a home or community, and this sense of comfort and connection thus facilitated their engagement with iOAT.

#### Trusting, supportive, non-judgemental relationship with staff

Given that iOAT service users access up to three clinically supervised injections a day, they have close and frequent interactions with service providers on a daily basis, in addition to other DPC staff members. The tone of these interactions has the potential to greatly affect clients’ care. All participants shared that their relationship with service providers is overwhelmingly positive and supportive. In particular, service users expressed their confidence to connect in a timely and meaningful way with service providers on site and felt assured by the providers’ ability to identify challenges or unmet needs. The site’s PCC approach to relationship building creates opportunities for open and inclusive dialogue between service users and the interdisciplinary team of service providers:

*Everything I had an issue with, [service providers] were able to help me with, right? And they had staff willing to take that on, and I didn’t have to really push them super hard, I just had to identify the problem. And I had to show up. That was my – that was the only thing that was required of me, was showing up. Right? And I was able to do that. And so, by being able to do that day after day, [service providers] were able to take things forward, and sort of build on what happened yesterday, and create some forward momentum on that.* (Service User Claude)

The atmosphere of positive, supportive interactions between service users and providers thus opened spaces for productive collaboration on care plans that lessened the treatment burden on service users and facilitated growth.

#### Barriers to iOAT integration that restrict PCC

Service providers reported that their ability to provide holistic PCC can be restricted by system-level barriers that arise from incongruencies between regulatory bodies (e.g. College of Pharmacists, provincial and federal governments) and a PCC approach. These tensions include inadequate funding for integrated care sites, restrictions on medication dispensation among community pharmacies, and restrictions on take-home iOAT doses. Specifically, while nurses lead iOAT programs in many sites, their role can be constrained by regulatory barriers. For instance, one provider described a regulatory inconsistency with take-home oral co-prescriptions, where service users could not take the medication off-site if it was dispensed from the DPC, but they could take the same medication off-site if it was dispensed by a community pharmacist. She argued that such disjunction in medication regulation can lead to a high treatment burden on clients and a loss of continuity of care:

*[The College of Pharmacists] basically didn’t want a nurse to be involved directly – the medication had to come right from a pharmacist to the patient […] it’s just kind of another barrier, because what you’re asking is the patient to now come here twice and go to the pharmacy, so we had people just drop off taking the [Kadian] which probably led to more illegal drug use.* (Service Provider Joan)

To expand iOAT flexibility so the treatment system meets clients’ needs, service providers suggested increased access to diverse iOAT formulations, access to unobserved doses (i.e., take-homes), and longer site hours. Regarding take-home iOAT, participants concurred: “[Take homes] would allow me to be more comfortable with when I wanted – needed to do it rather than, you know, by a certain time or whatever. […] Just having more control and feeling better about it” (Service User Corbin). Finally, beyond the increased flexibility, service users and providers routinely suggested hiring a primary care practitioner and a social worker ‘in-house’. Participant felt these expansions of care have great potential to improve the quality and scope of care that service users can access.

### A sense of place

Service users described the inclusion of iOAT within their care as finally receiving the medication they needed: “[The medication] actually works. It’s the first drug that I’ve seen that actually works” (Service User Dion). For service users engaging with the integrated care site for the first time, access to injectable DAM or HDM was their primary reason for engaging in care at the integrated care site. For others, it complemented their ongoing care in other on-site services, or their ongoing OUD care with oral opioid agonist treatment or fentanyl patches (iOAT is commonly co-prescribed with other OUD medications, particularly to maintain stability between injectable doses [13]. Our findings suggest that the support and structure already existent at the site, the accessibility of the location for its service users, the social relationships and community belonging, and the food program intersected with iOAT in a way that maintained engagement for clients accessing iOAT for the first time. From service users’ perspective, the DPC was geographically convenient to their place of residence, and near other medical and social services they accessed (e.g., HIV treatment at the nearby hospital), and was a space where clients felt comfortable socializing and building community. Thus, we refer to these place-specific factors as offering a particular sense of place that participants feel comfortable in.

#### Preferred physical location

A common place-specific factor identified by service users was the convenience of and appreciation for the integrated care site’s physical location. The site is uniquely located in a neighborhood that is removed from high levels of street entrenched substance use, in contrast to the other iOAT sites in downtown Vancouver. Service users expressed that this separation is beneficial because they can take space from the environments and stimuli that some associate with their previous substance use and may trigger anxiety. Further, service users indicated that the site’s proximity to their place of residence (e.g., long-term housing on-site, apartments nearby) and other medical services (e.g., HIV treatment at the hospital) was advantageous. As one service user, William, noted: “I used to live down on [neighborhood], OK? … No interest in spending time down there at all. I broke free of that, thank you very much.” When faced with the option of attending a new clinic during the pharmacy transition (albeit momentarily), service users continued to express their preference to remain at the DPC even when offered transport to the alternative iOAT site (e.g. bus tickets, cab vouchers). Service users associated the other neighbourhood with feelings of discomfort, lack of safety, and potential triggers, like Jay, who explained, “I wanted to avoid going through [the other neighborhood]. It’s not really something that I feel comfortable with.” Providers reiterated these perspectives:

*Well, people just didn’t understand why this had to happen and were frustrated. I think there were a lot of concerns of getting to [alternate site] because it’s so far, and they didn’t want to go there because of their previous experiences [of the neighbourhood]. One thing that is unique about this program is because it’s in the west end of Vancouver, so I think people like location-wise, especially for our older demographic, so, telling them that their option was [alternate site], they weren’t happy with that, so a lot of them just said no right away to that. (Service Provider Kim)*

Overall, the geographic location of the site promotes the possibility of forward momentum and acts as a demarcation between old habits and new patterns.

#### Social connection and community belonging

Most service users described the site as a social hub, and the ability for both casual and deep social interaction incentivizes their continued engagement in iOAT. Particularly, the site is an environment that enables peer interaction between individuals with similar life experiences (i.e., addiction) through conversation and/or joint recreation. As one service user, Raymond, stated simply, “I have lots of friends here.” Another service user expanded:

*Well, just in general, I’ve had some really good conversations with people here that I’ve never had with anyone ever, because they’re in the same place. They’ve been through the same - travelled the same roads –suffering in addiction.* (Service User Leo).

Beyond peer-to-peer social interaction, service users reported that the addition of iOAT to the integrated care site gave new service users a sense of community belonging that supported their self-determined wellness trajectory. Feeling at ease or ‘at home’ meant that service users were given a chance to feel rooted in a comfortable, welcoming space and with a group of people they felt deeply connected to:

*Here and […] are the only two places I feel comfortable, that I feel like people understand me. […] There’s love here, you know. You know, Doctor Peter was an amazing guy. […] The service that I get, food that we eat, just the way that we’re actually loved, you know what I mean? Like, cared for.* (Service User Dion)

Services providers similarly remarked on the warmth of the site environment:

*When you walk into the Dr. Peter Centre, there’s a calm inviting energy and vibe to the organization. You don’t feel rushed, you don’t feel out of place, you don’t feel stigmatized, quite the opposite, you feel welcome, you can come in, you can have food, you can participate in other stuff or not, whatever you want. So, there’s a stability, right? There’s a stability to our routine without judgment.* (Service Provider Jamie)

These expressions of comfort, care, and welcoming reported by both service users and providers denote a powerful sense of social and community belonging that facilitates iOAT engagement.

#### Food as a pathway to service engagement

In line with prior findings at this integrated care site, service users continuously reported that the meal program was a crucial anchor to iOAT. Food addresses service users’ most basic human need, which then poises them to access other site resources to meet their other needs (e.g., iOAT, counselling, recreation). Given the communal aspect of eating in a dining room, the food program facilitates socialization with peers and service providers. Once clients begin to attend the site regularly, participants shared that they are more likely to access the other available services and make decisions related to their health – an opportunity they may not have had for a long time. For example, William, a service user, stated, “Because with coming for that food every day you have, you know, a world of opportunities and resources available to you here.” Once food security is addressed, service providers can take their time in getting to know new service users at the site to begin forming therapeutic relationships, establish trust, and get a sense of what else might be needed.

*[The food] attracts people to come here, because we do know that these people are hungry. If they can get through the day with having their two meals a day here, which we provide enough calories to survive a day or more, through the two meals that we provide. … it’s a major point of engagement. Because now we’ve got them here and they’re sitting and they’re just like a regular person now, they’re in a dining room, and they’re having their lunch*, *… So that it gives us a chance, so we can actually go up and say hi, how you doing? Most people will come down, sit, take their time, have their meal, and talk amongst themselves, or get some other services from the counsellors […] The food draws them in for that*. (Service Provider Frankie)

The meal program thus serves both the primary function of meeting service users’ nutritional needs, which has direct health benefits, and a secondary function as a gateway to a broader network of health and social services (e.g., iOAT), therapeutic relationship building, and community.

## Discussion

Since its resurgence in the early 1990s, iOAT has been framed within a strict and regulated medicalized approach [47, 48]. Offering iOAT within an integrated service setting like the DPC opens possibilities to provide comprehensive, accessible addiction care across Canada. The present study explored how service users and providers experience the incorporation of iOAT within an integrated care site and the processes by which people remained engaged in iOAT. Our findings indicate that incorporating iOAT at an integrated care site occurred through a holistic approach to treatment/services and place-specific factors which embody a particular sense of place 19] where service users feel safe and comfortable.

The holistic approach to care includes key principles of PCC such as client autonomy and shared decision making which permit individualized treatment approaches and are core elements of person-centered addiction care [31, 33]. Particularly, client autonomy in making informed healthcare decisions is a tenant of ethical medical practices [46], and service providers worldwide are keen to embed client autonomy into their care provision [1, 30, 31]. At this integrated care site, service users shared that they felt no pressure or expectation to engage beyond the level they felt comfortable with, and they had autonomy to determine what their care looked like. Service providers agreed that autonomy was part of the organization’s mission values. At the same time, service users and service providers valued that services which respond to service users’ diverse needs, including iOAT, are consolidated at one site to provide both an entry to and extension of holistic care [11].

IOAT programs are typically highly structured and medicalized [20]. A key feature of the integrated care site’s holistic approach is that it de-medicalizes care. By distancing themselves from the discomfort, fear, and trauma that service users may have experienced in other healthcare settings [8], the integrated care site acts as a community first and a treatment site second. As a result, several barriers to accessing a highly medicalized treatment like iOAT decrease significantly. Service users also highlighted that their positive, non-judgemental, trusting relationships with service providers empowered them to make treatment progress. These types of supportive therapeutic relationships are also a core principle of person-centered substance use care [33]. Service users could have open dialogue about their goals and knew that service providers would respect their perspectives and choices.

The rigid regulations governing controlled substances have a long history of obstructing person-centered, individualized care [43]. System tensions continue to restrict the holistic approach at the DPC, as they do in other iOAT settings [31]. Regulatory and protocol demands are rigidly embedded within the DPC’s practices and hinder service providers’ ability to enact beneficial change in their service delivery. Service providers felt held back by restrictions and confusion that pharmacy regulations imposed on care. By imposing regulations that prevent service users from accessing the medication that attracts them into care, “the international drug control system presently stands in the way of a public health approach” [44, p. 235]. Service users also expressed that the schedule of daily supervised doses is inaccessible, and that take-homes doses are a desired expansion of treatment. The rigid schedule of daily site visits is a pervasive barrier in iOAT programs, and findings indicate that take-home doses are indeed a viable option to increase iOAT clients’ quality of life and continuity of care [38, 39]. Further, service users expressed that having an in-house general practitioner and access to different medication formulations are changes that would improve the individualization, autonomy, and comprehensiveness of their care. These recommendations align with a recent study which found that iOAT nursing staff desire multi-disciplinary addiction healthcare teams and greater capacity to provide individualized treatment and equitable care access [5].

IOAT engagement was dominantly sustained by a sense of place, which refers to the community and atmosphere at the site that facilitated a feel deep connection between the site and the service users. Place is a key factor in healthcare considering the importance of contextual factors and risk environments as determinants of health broadly, and substance use disorders specifically [12, 14]. While macro-level places (e.g., countries, neighbourhoods) can impact peoples’ health and substance use [10], more micro-level places (e.g., SROs, healthcare sites) can either limit or facilitate peoples’ health [28]. Integrated care sites such as the DPC can thus be understood as an ‘enabling place’ that promotes clients’ achievement of their self-identified goals, particularly through social and affective resources [17]. Given the stigmatizing interactions injection drug users frequently encounter in traditional healthcare spaces, connection to a non-stigmatizing place can be deeply impactful [42].

A core element of service users’ place-specific connection to this integrated care site, and a crucial anchor that kept service users engaged in iOAT, was the meal program. The social and community attachment engendered by food aligns with prior research on the DPC’s food program and highlights the emerging importance of nutrition in OUD care [9, 35]. Communal mealtimes provided the opportunity to form social routines with both peers and service providers. Additionally, the meal program functioned as a low barrier entry point into the site’s wider suite of supports. Service users also shared that they valued the social atmosphere of the site, as they share spaces with non-judgemental peers with whom they can form friendships with through shared life experiences. The opportunity for socialization forms an overwhelming sense of community belonging at the DPC [36]. Service users shared that they felt comfortable, loved, and at home. Finally, the DPC’s physical location was crucial to maintain iOAT engagement. Service users valued that the site was in an area outside of the street-entrenched substance use so they could distance themselves from triggering environments. The geographic location of the site enables forward momentum towards new goals rather than past traumas. This finding underscores the need to expand iOAT to diverse neighbourhoods and regions, particularly to reach remote Indigenous communities (Ominika, 2021). Given our results, regions that do not currently offer iOAT but have offer other integrated substance use care might consider embedding iOAT in those settings as the first stage of iOAT implementation. Capitalizing on existing infrastructure might reduce the barriers, particularly related to cost, of brining iOAT to regions that do not currently embrace harm reduction approaches.

With iOAT sites emerging across Canadian provinces [18], healthcare systems must ensure they are flexible and adaptive to the diverse needs of the population they aim to serve. Regulatory bodies should support the provision of iOAT within the full continuum of care and not strictly as a specialized treatment. Our overall findings suggest that incorporating iOAT within an integrated care site as part of comprehensive wraparound supports offers clients autonomy, convenience, comfort, community, and support in their OUD treatment. Integration facilitates PCC and an individualized approach to OUD treatment. Future research might build upon our qualitative findings by assessing client outcomes (e.g., mental and physical health, street substance use) on iOAT in integrated care settings vs. in isolated clinical settings.

### Limitations

While recruitment aimed to capture clients with diverse identities, it is of note that all the service user participants were men with an average age of 57.36 years, and the majority identified as white. There is still much to do to engage clients of diverse genders, races, and ages who would benefit from iOAT access through integrated care. Further, despite our recruitment attempts, the research team was unable to connect with any former iOAT service users at this site. The perspectives of former clients would be extremely valuable in identifying and exploring areas warranting improvement when embedding iOAT within integrated care sites. It is important to recognize that despite growth in the provision of person-centered addiction care, such advancements are still subject to ongoing improvement.

## Conclusion

Our findings attest to the need for highly regulated and medicalized treatment approaches to be reconsidered and instead evolve to meet the needs of services users and the providers who support them. This exploratory study provides evidence for policy makers and stakeholders to change regulations so that they foster and facilitate the integration of iOAT into less medicalized, isolated, and regulated sites.

### Supplementary Information

**Additional file 1.** Service user and provider semi-structured interview guides.

## Data Availability

The datasets generated and/or analysed during the current study are not publicly available due to the qualitative design that risks potential reidentification as well as to protect the privacy of clinical patient data, but a modified version of the data may be available from the corresponding author on reasonable request.
