# Supplementary material for: “It feels like I’m coming to a friend’s house”: an interpretive descriptive study of an integrated care site offering iOAT (Dr. Peter Centre)
Source: Addict Sci Clin Pract. 2023 Dec 2;18:73. doi: 10.1186/s13722-023-00428-4 (PMC10693115; doi:10.1186/s13722-023-00428-4)
Supplement: Supplementary file 1 — Additional file 1. Service user and provider semi-structured interview guides. [file 13722_2023_428_MOESM1_ESM.docx]

iOAT in Integrated Care Settings – Interview Guide

**Topic Guide for Service Users**

Thank you for agreeing to participate in this interview. We are interested in learning about your perspectives and experiences with injectable opioid agonist treatment (iOAT) within an integrated health care setting.

We also would like to take this moment to explain some of the outcomes of these interviews. As researchers, we are interested in hearing about iOAT services, including whether the ways it is or was provided have worked or not worked for you. We will also be asking you about changes that would make it work better for you. That said, as researchers we do not hold the power to change policy or programs. We aren’t policy makers or government officials so while we hope that this study will help policy makers and other officials design programs that best meet your needs, we can’t guarantee that it will. We will make every effort to pass all we learn from this study to those who are in a position to implement changes.

We can guarantee confidentiality from the research team, as per our guidelines. We would like to record this conversation and transcribe it so that we can listen to it and ensure our interpretations are accurate. Only the research team will have access to the recordings and transcriptions.

**START RECORDING**

Questions for participants:

1. What were your thoughts/impressions when iOAT was implemented at [insert name of integrated health care facility e.g., Dr. Peter’s Centre]? PROBES: Treatment at the time when iOAT was implemented; previous treatment history with iOAT; how were the first days?

***The aim of this first question is to build a trajectory of the client’s experience with iOAT at the integrated health care site from before having iOAT (if applicable), at the moment they knew iOAT was coming (if applicable), the first days of iOAT and now. We build this trajectory from the client, allowing them to anchor themselves in their own experience and decide where their ‘trajectory’ starts.

2. What other services were you accessing at [insert name of integrated health care facility] and how were things going?  PROBES: Activities engaged in at the site, do you use other services here, what are the things you use the most at the site? What services or activities are you accessing somewhere else?

*** The aim of this question is to get a sense of the integrated care experience at the integrated health care facility and elsewhere.

**3. Thinking about how you access iOAT here, how does it influence your overall health and wellness?** PROBES: What changes have or have not occurred in your daily life? Have you experienced any changes in treatment experiences? Changes in social connections?

Further probe on lateral stigma: Have the relationships with the other people coming changed (i.e, new people to iOAT and former people you already knew)?

**4. How are your relationships with other members/clients at the center?** PROBES: those that access iOAT; new iOAT clients; those that do not access iOAT.

Further probe on tensions: How have the relationships changed? How has your connection to the center been (or not been) impacted by these relationships and dynamics?

*** The aim of this question is to capture the potential lateral tensions that arise due to inequities in the distribution/access to iOAT and other services.

**5. How has your connection to the site been impacted (or not been impacted) by the integration of iOAT in general?**

**PROBES:** Connections with staff, providers, people outside the center you’re hanging out more (or less) with, overall community? Sense of place and neighbourhood?

Note: These are broad questions that as the theory evolve will become more specific (iterations within the grounded theory approach). Probes refer to examples of areas to explore base to the study research question.

iOAT in Integrated Care Settings – Interview Guide Round Two

**Topic Guide for Service Providers**

Members at the DPC receiving diacetylmorphine were subject of a change due to pharmacy logistics. As a result, for a one-month (approx.) period members were given a choice and accommodation to either switch to HDM at the DPC or be transferred to DAM at Crosstown. This transition period is unique for many people accessing iOAT, and it could support service users and providers to describe how a member made their choice (e.g., medication and site which they inject). As program struggle with regulatory changes, we want to be able to define how these place-specific factors (e.g., food, community, staff, location, etc.) interplay with the medication that they

Thank you for agreeing to participate in this interview. We are interested in learning about your perspectives and experiences with injectable opioid agonist treatment (iOAT) within an integrated health care setting.

We also would like to take this moment to explain some of the outcomes of these interviews. As researchers, we are interested in hearing about iOAT services, including whether the ways it is or was provided have worked or not worked for you. We will also be asking you about changes that would make it work better for you. That said, as researchers we do not hold the power to change policy or programs. We aren’t policy makers or government officials so while we hope that this study will help policy makers and other officials design programs that best meet your needs, we can’t guarantee that it will. We will make every effort to pass all we learn from this study to those who are in a position to implement changes.

We can guarantee confidentiality from the research team, as per our guidelines. We would like to record this conversation and transcribe it so that we can listen to it and ensure our interpretations are accurate. Only the research team will have access to the recordings and transcriptions**.**

**START RECORDING**

1. Can you tell me a little bit about your role at the Dr. Peter Center? PROBE: More specifically, what is your role within the DPC’s iOAT program? (If we have interviewed this person in the past, we tag this question to the next)

Probes: What services / medications do you provide to the program? How long have you been in this role? Have there been any challenges / obstacles in the fulfillment of your role?

1. Can you tell me a little about the recent transition of iOAT medications at the Dr. Peter Center?

Probes: What was your role then? What was that process like? What were some of the challenges? How were members supported in this transition? Did you see member care be affected by this transition? If yes, how so?

1. Continuing building from the perspectives given in point 2, How the integrated care facility handled this transition? How was the interplay with the medications was handled and perceived as providers? Were there key aspects of the integrated facility, the way iOAT is provided, that as a service user, you think was key on maintaining continuation of care, for example?

Probes: Do you have any examples? Do you find, in your role, any services that might have been key to support members and were not present?

1. In your view, what could be done at a regulatory level (PROBE: specific actions) to support service providers, like you, during these transitions to provide iOAT within a person-centered/individualized care framework?
